# 

**Published:** 1890-07-19

**Authors:** 


SATURDAY, JULY 19th, 1890.

**Is Marriage a Sin?** - - - - - - - - 225

**Words of Consolation.**—Prayer for Recovery - - - 226

**The Doctor's Pulpit.**—On the March - - - - - 227

**The History and Capabilities of Herbal Simples.**—By W. T. Fernie, M.D. XV.—Celandine—Celery - - - 228

**Before the Lords' Committee.** VII.—Nursing at the London Hospital - - - - - - - - - - 229

**Everybody's Page** - - - - - - - - - 231

**Notes and News** - - - - - - - - - 232

**Annotations.**—The Kyrle Society—Farm Houses for Summer Holidays—An Open Door - - - - - - - - - 234

**General Charities** - - - - - - - - - 239

**The Practitioner's Mirror and Retros_ ct:**—

Medicine—Malarious Hæmophilia—Ureametry—Nature of Phthisis, 235; Sudden Deaths in Laryngeal Disease - - 236

Surgery—Unreduced Dislocations of the Femur, 236; Ovariotomy - - - - - - - - - - - - 237

New Drugs, Appliances, and Things Medical—The Excelsior Bed Lift (Monkhouse's Patent)—Caffyn's Liquor Carnis—Salvine Dentifrice—The Duplex Instanter Thermometer - - - - - - - - - - 238

**Spelthorne Sanatorium** - - - - - - - - 239

**Passing Topics.**—Probationer Nurses - - - - - 240

**Scraps and Gleanings** - - - - - - - - 240

### EXTRA SUPPLEMENT—THE NURSING MIRROR.

**En Passant.**—Wolverhampton Institution—Torbay Hospital—Philadelphia School—Short Items—The Hospital Commission—Workhouse Nurses—Nightingale Nurses - - lxvii
**Sketches of Insanity.**—By Francis H. Walmsley, M.D. III.—The False Beliefs of the Insane - - - - - - lxviii
**An Appeal to the Humanity of the Public** - - lxix
**The Nurses' Bookshelf.**—The Registration of Nurses - lxix
**Everybody's Opinion.**—Once in a Lifetime—A Plea for Cheap Tickets—A Modern Miracle - - - - - - - lxx
**Vacancies** - - - - - - - - - - lxx
**Notes and Queries** - - - - - - - - lxx
**Amusements and Relaxation** - - - - - - - lxx

